# Unruptured Cerebral Aneurysms: Evaluation and Management

**DOI:** 10.1155/2015/954954

**Published:** 2015-06-04

**Authors:** Norman Ajiboye, Nohra Chalouhi, Robert M. Starke, Mario Zanaty, Rodney Bell

**Affiliations:** ^1^Department of Neurological Surgery, Division of Neurovascular Surgery and Endovascular Neurosurgery, Thomas Jefferson University Hospital, Philadelphia, PA 19107, USA; ^2^Department of Neurological Sciences, Division of Neurocritical Care, Thomas Jefferson University Hospital, Philadelphia, PA 19107, USA; ^3^Department of Neurosurgery, University of Virginia, Charlottesville, VA 22903, USA

## Abstract

The evolution of imaging techniques and their increased use in clinical practice have led to a higher detection rate of unruptured intracranial aneurysms. The diagnosis of an unruptured intracranial aneurysm is a source of significant stress to the patient because of the concerns for aneurysmal rupture, which is associated with substantial rates of morbidity and mortality. Therefore, it is important that decisions regarding optimum management are made based on the comparison of the risk of aneurysmal rupture with the risk associated with intervention. This review provides a comprehensive overview of the epidemiology, pathophysiology, natural history, clinical presentation, diagnosis, and management options for unruptured intracranial aneurysms based on the current evidence in the literature. Furthermore, the authors discuss the genetic abnormalities associated with intracranial aneurysm and current guidelines for screening in patients with a family history of intracranial aneurysms. Since there is significant controversy in the optimum management of small unruptured intracranial aneurysms, we provided a systematic approach to their management based on patient and aneurysm characteristics as well as the risks and benefits of intervention.

## 1. Introduction

Intracranial saccular aneurysms are acquired vascular abnormalities that cause outpouching of the arterial wall. They are often located at the bifurcation of the arteries in the anterior circulation of the Circle of Willis. There has been increased detection of unruptured intracranial aneurysms in clinical practice due to the frequent use of CT and MRI [1]. Therefore, in this review, we present a comprehensive overview of unruptured intracranial aneurysm with special regards to the predictors of rupture, the risks of medical management in comparison to clipping and coiling. We also provide family screening recommendations in patients with unruptured intracranial aneurysm. Furthermore, we discuss new endovascular techniques like flow diverting stents and their current indications.

## 2. Epidemiology

Intracranial aneurysms occur in 1-2% of the population and account for about 80–85% of nontraumatic subarachnoid hemorrhages [1]. Autopsy studies indicate prevalence in the adult population between 1% and 5%; however, 50% to 80% of all aneurysms do not rupture during the course of a person's lifetime [2]. Unruptured intracranial aneurysms are more common in women with a 3 : 1 ratio of women to men [3]. Intracranial aneurysms are sporadically acquired lesions; however, a rare familial form has been associated with conditions like autosomal dominant polycystic kidney disease, Marfan's syndrome, Ehlers-Danlos syndrome type IV, fibromuscular dysplasia, moyamoya disease, sickle cell disease, and arteriovenous malformations of the brain [4, 5]. Approximately 5% to 40% of patients with autosomal dominant polycystic kidney disease have intracranial aneurysms, and 10% to 30% of patients have multiple aneurysms [4, 5]. An important risk factor for aneurysm is a family history. Patients with one affected family member have approximately a 4% risk of having an aneurysm, whereas patients with 2 or more affected first-degree family members have a 8%–10% risk of having an aneurysm [6]. Current guidelines recommend screening with intracranial magnetic resonance angiography for people with two immediate relatives with intracranial aneurysms and for all patients with autosomal dominant polycystic kidney disease [7]. The modifiable factors that may increase the risk for aneurysmal SAH include smoking, alcohol use, and hypertension [7]. The estimated incidence of subarachnoid hemorrhage (SAH) from a ruptured intracranial aneurysm in the United States is approximately 6–10/100,000 person-years [8]. Approximately 5% to 15% of cases of stroke are related to ruptured intracranial aneurysms [8]. Subarachnoid hemorrhage is more common in women than in men (2 : 1) with the peak incidence occurring in persons 50 to 60 years old [9]. The fatality rate for SAH is 30%–40%, and as high as 3 in 5 of those who survive SAH may be functionally dependent [6].

## 3. Pathology and Pathophysiology

The genetic etiology of intracranial aneurysm is complicated as demonstrated by a large meta-analysis of genetic studies which identified 19 single nucleotide polymorphisms associated with sporadic intracranial aneurysm. The strongest associations were found on chromosome 9 within the CDKN2B antisense inhibitor gene, on chromosome 8 near the SOX17 transcription regulator gene, and on chromosome 4 near the EDNRA gene [9]. Hypertension and smoking-induced vascular changes are involved in the process by which aneurysms form, grow, and rupture [4, 5]. The most common histologic finding is a decrease in the tunica media, the middle muscular layer of the artery, causing structural defects. These defects, combined with hemodynamic factors, lead to aneurysmal outpouchings at arterial branch points in the subarachnoid space at the base of the brain [10].

## 4. Natural History

Understanding the natural history of aneurysms is important in making treatment decisions. The International Study of Unruptured Intracranial Aneurysms (ISUIA) was a prospective cohort study that followed 1,692 patients with unruptured aneurysms that were 2 mm or larger (1,077 without prior history of SAH). ISUIA documented an overall annual aneurysmal rupture risk of 0.7% [12]. Two important factors in predicting risk of rupture include size and location. Various studies have shown that larger aneurysms have the greatest risk of rupture. However, other factors that can influence rupture risk include aneurysm location and patient factors such as age younger than 50 years, those with hypertension, and those with multiple aneurysms [12].

A Japanese prospective study reported the natural history of patients with aneurysms 3 mm or larger with an annual rupture rate of 0.95% [14]. Furthermore, the risk of rupture increased with size, with a significant increase for aneurysms 7 mm or larger. Other risk factors for rupture included location on the anterior or posterior communicating artery and presence of a daughter sac [14]. A large meta-analysis from currently available literature demonstrates that other factors including age over 60 years, female sex, Finnish or Japanese descent, aneurysm size over 5 mm, posterior circulation location, and symptomatic unruptured aneurysms have a higher risk of rupture [26].

Findings from many retrospective studies have suggested that rupture risk is reduced in patients taking aspirin [27, 28]. However, it remains unclear whether the benefit of aspirin use in patients presenting with an unruptured intracranial aneurysm outweighs the potential risks, and a randomized double blinded clinical trial will be needed to answer this question with more certainty [29]. In a study of 747 consecutive patients with intracranial aneurysm presenting at a single hospital, the rate of hemorrhage was higher among those not taking aspirin (40%) than among those taking aspirin (28%), but the overall morbidity and mortality outcome of those experiencing subarachnoid hemorrhage was not affected by aspirin use [30]. In a case-control study of 1797 incident cases of intracerebral hemorrhage and subarachnoid hemorrhage, aspirin use was associated with a decreased risk of subarachnoid hemorrhage (odds ratio 0.82, 95% CI 0.67–1.00) compared with no aspirin use [31]. In an analysis of data from the ISUIA untreated cohort, patients who used aspirin most frequently had the lowest risk of aneurysm rupture during follow-up [32].

There are limited natural history data available for patients with familial intracranial aneurysm. ISUIA did not show that a family history was predictive of hemorrhage in a regression analysis. However, in the familial intracranial aneurysm study, 548 first-degree unaffected relatives of people with a familial history of intracranial aneurysm had MRA screening: 113 participants had 148 unruptured intracranial aneurysms, 5 of whom had an unruptured intracranial aneurysm that was 7 mm or larger in diameter. Two patients had aneurysmal rupture during follow-up, which represented an annual rupture rate of 1.2 per 100 patients (95% CI 0.1–4.3). The rupture rate in this cohort was 17 times higher than that for patients with an unruptured intracranial aneurysm in ISUIA after matching for aneurysm size and location. However, the small number of ruptures and large 95% CI precluded definitive conclusions regarding rupture rates in familial aneurysm [33].

Aneurysms presenting with subarachnoid hemorrhage tend to bleed again at a rate of 9% within the first 72 hours after the initial episode [34]. Therefore, patients with known intracranial aneurysms presenting with cranial-nerve palsies or brain-stem dysfunction should be evaluated and treated promptly because of the increased risk of rupture of 6% per year [35].

In a recent article, Greving et al. [11] proposed a practical risk score called PHASES score. It is used to predict a patient's risk of aneurysmal rupture based on population (geographical location), hypertension, age, size of aneurysm, earlier subarachnoid hemorrhage from another aneurysm, and site of aneurysm. These predictors were selected based on a systematic review of and pooled analysis from 8382 participants in 6 prospective cohort studies with subarachnoid hemorrhage as outcome (readers should refer to Tables 1 and 2).

## 5. Clinical Presentation

Unruptured intracranial aneurysms may be incidental findings as a result of complaints unrelated to the aneurysm or detected as they grow and cause compression on adjacent brain structures [32]. Such compressions include middle cerebral artery aneurysms causing hemiparesis, visual field defect, or seizure, posterior communicating artery or basilar artery aneurysms causing third cranial nerve palsy, cavernous sinus aneurysms causing a cavernous sinus syndrome, basilar distribution aneurysms causing compression of the brainstem, and, on rare occasions, an embolus from the aneurysmal sac causing transient ischemic attack or cerebral infarction due to distal embolisation [33]. Other cranial nerves can be involved, including trochlear and abducens nerves and the first division of the trigeminal nerve [36].

## 6. Diagnosis

The radiographic studies available to delineate the size and morphologic features of an intracranial aneurysm are CT angiography (CTA), magnetic resonance angiography (MRA), and angiography by direct intra-arterial catheterization (catheter angiography), which is considered the gold standard [37].

Several studies have evaluated the accuracy of detecting intracranial aneurysms by comparing CTA, MRA, and catheter angiography [38]. CTA uses thin-section contrast-enhanced CT with the aid of software generated images to show cerebral vessels in three-dimensional views. These reconstructed images can be generated in a few minutes, and they allow evaluation of the vasculature in close relation to the brain and the bones of the skull base, therefore facilitating surgical planning [39]. Sensitivities of CTA range from 77% to 97% and specificities range from 87% to 100% [40]. Sensitivity for aneurysms smaller than 3 mm drops to a range of 40% to 91% [41]. The role of CTA is very limited in patients with impaired renal function, since a large bolus of contrast material is often administered. MRA is highly sensitive and specific for the detection of intracranial aneurysms. MRA has a sensitivity of 70% to 99%, and a specificity of 100% for aneurysms 3 mm or greater in diameter; however, the sensitivity diminishes for very small aneurysms (under 3 mm in diameter) to as low as 40% [42]. MRA is more difficult to use in critically ill patients because it takes considerably more time to perform.

Catheter angiography, with or without the advanced three-dimensional capability, is the gold standard which provides detailed evaluation of the aneurysm in its relation to other vessels. However, catheter angiography is more expensive and invasive than either MRA or CTA. Its risks, even in the hands of experienced operators, include neurologic complications occurring in 1.0% to 2.5% of cases, with permanent impairment in 0.1% to 0.5% [43]. Other associated risks include femoral-artery injury (0.05% to 0.55%), groin hematoma (6.9% to 10.7%), and adverse renal effects induced by contrast material (1% to 2%) [43].

Overall, brain MRA or CTA are the methods of choice for screening of unruptured aneurysms because of their noninvasive nature; however, a cerebral angiogram is sometimes used to better clarify the details of an aneurysm. It should be noted that the ability of MRA and CTA to provide such morphological data is rapidly improving [44]. For those patients in whom contrast administration is contraindicated (including patients with renal failure in whom MRI-related gadolinium contrast is contraindicated), MRA is the screening method of choice because gadolinium contrast administration is not needed [45]. In 10% of cases of subarachnoid hemorrhage, cerebral angiogram may not detect any aneurysm. In cases in which the angiography is negative, it should be repeated in one to six weeks [46]. The proposed mechanism for the cause of subarachnoid hemorrhage without an identified aneurysm is hypertensive rupture of a small artery or vein. In these circumstances, an MRI of the brain and cervical spine with and without gadolinium should be obtained to rule out a thrombosed aneurysm, spinal arteriovenous malformation, dural arteriovenous fistula, or hemorrhagic tumor; however, the diagnostic yield is small [47, 48].

## 7. Management

Optimum management of an unruptured intracranial aneurysm should involve the comparison of the risk of aneurysmal rupture without any intervention with the risks of surgical clipping or endovascular treatment. The factors that should be considered include (1) aneurysmal factors, such as location, size, morphology, whether a thrombus exists within the aneurysm, and the presence of a daughter sac or multiple lobes, and (2) patient factors such as age, medical history, history of subarachnoid hemorrhage, and family history of subarachnoid hemorrhage [49].

There are three management options for unruptured cerebral aneurysms: conservative management, surgical clipping, or endovascular treatment. Currently, there is a lack of prospective randomized controlled trials to guide therapy, particularly in comparing intervention with conservative management. Many published articles are retrospective in nature, and they lack objective short- and long-term assessment of outcomes [50].

Currently our best information regarding management of unruptured aneurysm is based on observed rates of complications in aneurysm treatment compared to the natural history of unruptured aneurysm [50].

### 7.1. Conservative Management

Conservative management is usually recommended for patients over the age of 60 years and for small (<7 mm) aneurysms, except in those with a strong family history of subarachnoid hemorrhage or a symptomatic aneurysm [51]. It should be noted that for patients with aneurysms of 7–12 mm in diameter, management is individualized, but many elderly patients with aneurysms in the anterior circulation can be considered for conservative management. For patients over the age of 60 years with aneurysms greater than 12 mm in diameter, an interventional procedure should be considered, while considering the patient's overall health status and the presence of factors that might increase surgical or endovascular risks [51].

It is imperative that all patients treated conservatively should be counseled about potential risk factors (such as hypertension and tobacco use) for aneurysm growth and rupture. Therefore, hypertension should be aggressively controlled, and smoking cessation should be strongly advocated for all patients who smoke. Alcohol should be used only in moderation.

Conservative management consists of routine periodic follow-up imaging with MRA or CTA and physician visits to review the studies. There is no recommended optimum interval, but a reasonable approach would be to repeat the MRA or CTA on an annual basis for about 3 years and then on several further occasions at a reduced frequency [44]. With regards to small unruptured and asymptomatic aneurysms of 2-3 mm in diameter, less frequent imaging can be performed if repeat imaging at 1 and 2 years shows stability of the aneurysm. Patients with aneurysm growth should be strongly considered for interventional treatment [45]. In a recent study using MRA to evaluate 173 unruptured aneurysms over a 4-year period, size at initial detection was a key predictor of aneurysm growth [44]. The overall frequency of aneurysm growth was 6.9% for aneurysms less than 8 mm in diameter, 25% for aneurysms 8–12 mm in diameter, and 83% for those greater than 12 mm in diameter [44]. Other predictors of aneurysmal growth demonstrated by a study which used MRA to assess 130 patients with 159 aneurysms in which 14 aneurysms grew included middle cerebral artery location, presence of more than one aneurysm, and aneurysm size greater than 4 mm [45]. The risk of aneurysm rupture after confirmation of aneurysm enlargement is not known with certainty. In a study in which CTA was used to evaluate 165 patients with 258 aneurysms, 18% of aneurysms grew [37]. The rate of aneurysmal rupture was 2.4% per patient-year in aneurysms with growth and 0.2% in those without growth. Independent predictors of aneurysm growth were initial size of aneurysm and tobacco use [37]. A recent prospective Finnish cohort study by Korja et al. concluded that about 30% of all unruptured intracranial aneurysms rupture during a lifetime, and the risk factors for lifetime subarachnoid hemorrhage included current smoking, female sex, and aneurysm size of ≥7 mm in diameter [51].

There are few studies regarding the safety of antiplatelet agents and anticoagulants in patients with unruptured aneurysms. A Danish population-based case-control study demonstrated an increased association between SAH and dipyridamole use and new aspirin use, but not long-term aspirin use [27]. A case-control study utilizing the International Study of Unruptured Intracranial Aneurysms (ISUIA) suggested that aspirin use may have a protective effect against rupture and use of aspirin prior to rupture does not appear to worsen outcomes from SAH [15]. However, the use of anticoagulants has been associated with a poorer outcome from subarachnoid hemorrhage but not clearly associated with an increased risk for aneurysm rupture [29]. In patients with unruptured aneurysm, it is recommended that antiplatelet agents be used based on the patient's specific indication for such medication, while anticoagulants may be necessary due to specific indications; however, there should be careful consideration of risks and benefits and a thorough discussion with the patient.

### 7.2. Microsurgical Clipping

Microsurgical clipping requires access to the aneurysm via an open craniotomy. The aneurysm is dissected out and a tiny metallic clip is placed at the neck to isolate the aneurysm from the parent blood vessel. Surgical clipping is effective with complete occlusion obtained in greater than 90% of cases [46].

Surgical morbidity and mortality data are available from meta-analyses and prospective studies. Findings from one meta-analysis of 733 patients revealed a mortality rate of 1.0% and a major morbidity rate of 4% [49]. In a different meta-analysis of 2460 patients, a mortality of 2.6% and morbidity of 10.9% were reported; however publication bias was a limitation of this study [50]. Prospective data which was obtained from the International Study of Unruptured Intracranial Aneurysms (ISUIA) revealed a mortality of 2.3% at 30 days and 3.0% at 1 year for 798 patients undergoing surgical clipping [15]. Combined morbidity (which included substantial functional disability or severe cognitive impairment) and mortality from ISUIA was 17.5% in patients without previous intracranial hemorrhage at 30 days after surgical clipping and 13.6% in those patients with a previous hemorrhage from some other aneurysm [15]. Findings from a larger cohort of 1917 prospectively evaluated patients from ISUIA demonstrated a combined morbidity and mortality at 1 year of 12.6% for those without previous hemorrhage (death was 2.7%; functional disability only was 1.4%; impaired cognitive status was 5.5%; and both functional disability and impaired cognitive status was 2.8%) and 10.1% for those with previous subarachnoid hemorrhage from some other aneurysm (death was 0.6%; functional disability only was 0.9%; impaired cognitive status was 7.1%; and the status of both functional disability and impaired cognitive was 1.5%) [15]. Overall, morbidity and mortality were the highest in patients older than age of 50 years and with aneurysms that were large or in the posterior circulation [15]. The risks of microsurgical clipping include hemorrhage (0.25%), incomplete occlusion (5%), and recurrence (1.5%) [49].

In a recent meta-analysis by Kotowski and colleagues, clipping of unruptured intracranial aneurysms was associated with a mortality of 1.7% and a morbidity of 6.7% from a systematic review of 60 studies with 10,845 aneurysms in 9845 patients [19]. In this meta-analysis, morbidity rates were noted to be higher in large aneurysms (>25 mm) or posterior circulation aneurysms (readers should refer to Table 3).

### 7.3. Endovascular Management

The different endovascular techniques include (1) packing the aneurysm with coils, with or without adjunct techniques such as balloon inflation or stent placement at the aneurysm neck for more difficult cases; (2) use of flow diverting stents; and (3) use of liquid embolic agents.

The most common form of endovascular management is the deployment of the detachable coils into the aneurysm via microcatheter. These coils cause local thrombosis and isolation of the aneurysm from the parent artery. Patients that are ideal candidates for the use of coils are aneurysms with a narrow neck (<4 mm) and low dome-to-neck ratio (<2) [52]. Adjunct techniques such as balloon inflation or stent placement at the aneurysm neck are increasingly used in some of the more difficult cases such as wide neck (>4 mm) or high dome-to-neck ratio (>2) [20]. Adjunctive techniques for coil embolization prevent coils from protruding through the aneurysm neck into the parent artery, therefore reducing the risk of thromboembolic complications.

Flow diverting stents such as pipeline embolization device (Covidien, Irvine, CA) are indicated for large unruptured saccular or fusiform intracranial aneurysms (>10 mm) of the anterior circulation from the petrous segment to the superior hypophyseal segment [20]. Pipeline embolization devices (Covidien, Irvine, CA) consist of tightly braided mesh that allow flow into vessel branches, but cases stagnation of blood in the sac which results in the occlusion of the aneurysm [20]. Patients need to be pretreated with dual antiplatelet therapy (aspirin and clopidogrel). The recommendation is for the patient to be on lifelong daily aspirin (81–325 mg) and clopidogrel therapy is continued for a duration of 3 to 6 months after procedure [20]. The results of a multicenter retrospective study of the pipeline embolization device were recently published showing a 30-day morbidity and mortality of 6.3% and a long-term neurologic morbidity and mortality of 8.4% [53]. The morbidity and mortality were the highest in the posterior circulation group at 16.4% and the lowest in the internal carotid artery aneurysm (with size <10 mm) group at 4.8% [53]. The pipeline embolization study included a total of 793 patients with 906 aneurysms; when patients with ruptured aneurysm (9% of cases) were excluded, the overall morbidity and mortality for unruptured aneurysms was 5.7% [53].

Onyx HD-500 (ev3 Neurovascular) is an embolic agent that is indicated for large, saccular, wide necked aneurysms that are not amenable to surgical clipping or endovascular coiling. However, the use of liquid embolic agents (Onyx HD-500; ev3 Neurovascular) for large and giant intracranial aneurysms has declined due to higher risk of unfavorable outcomes. In a systematic review of the literature on the safety of endovascular treatment of unruptured aneurysms by Naggara and colleagues, procedure related poor outcomes occurred in 4.7% of patients, with higher risk of 8.1% in the subset of patients that received liquid embolic agents [20]. Cerebral Aneurysm Multicenter European Onyx (CAMEO) Trial was a prospective observational study that evaluated the use of Onyx in 97 patients with 100 aneurysms and it demonstrated a permanent neurologic morbidity of 8%, procedure related morbidity of 2%, and a delayed parent vessel occlusion rate of 9% [54].

Data from meta-analysis of existing literature suggests that the risk of unfavorable outcomes from endovascular management is approximately 4% to 5%, with a risk of mortality of 1% to 2% [20]. The 1-year morbidity rate was 6.4% and the mortality rate was 3.1% in 451 patients treated with endovascular coiling in the ISUIA study [12, 15]. Of note, the baseline characteristics of the endovascular group were different from the surgery group (which included older patients, larger aneurysms, and larger number of posterior circulation aneurysms); therefore, the results are not directly comparable. The risk of poor outcome with endovascular procedure was higher with aneurysm diameter greater than 12 mm and posterior circulation location.

There are no randomized clinical trial data that directly compare surgical clipping of aneurysm with coiling of unruptured aneurysms. Hwang and colleagues recently performed a meta-analysis of 24 studies to compare the effects of endovascular clipping and neurosurgical clipping in 31,865 patients with unruptured intracranial aneurysm [55]. Hwang et al. concluded that coiling was superior to clipping in the short term (≤6 months) in terms of disability and complications [55]. They demonstrated that a higher disability with clipping using the Glasgow Outcome Scale (OR, 2.38; 95% CI, 1.33–4.26) and Modified Rankin Scale (OR, 2.83; 95% CI, 1.42–5.63) in comparison to coiling and clipping resulted in higher disability in the short term (≤6 months) (OR on the Glasgow Outcome Scale, 2.72; 95% CI, 1.16–6.34), but not in the long term (>6 months) (OR for Glasgow Outcome Scale, 2.12; 95% CI, 0.93–4.84) [55]. Furthermore, clipping had 2.5 times more neurological and cardiac complications than coiling since the odd ratios (ORs) for neurological and cardiac complications were higher with clipping (1.94 with a 95% confidence interval [CI] of 1.09–3.47 and 2.51 with a 95% CI of 1.15–5.50) [55]. The limitations of the study by Hwang include the use of only observational studies and the study did not evaluate outcomes based on size and locations of aneurysms.

The risks of endovascular management include those associated with catheter angiography such as groin hematomas, infection, reactions to contrast material, and pseudoaneurysms [56]. Other risks include thromboembolism (2.5%), arterial dissection (0.7%), and parent artery occlusion (2%) [22]. Aneurysms treated with coils may require further treatment due to partially coiled aneurysm which may tend to recur and hemorrhage [22] (readers should refer to Table 4).

### 7.4. Clipping versus Coiling versus Conservative Management

**Figure 1:**
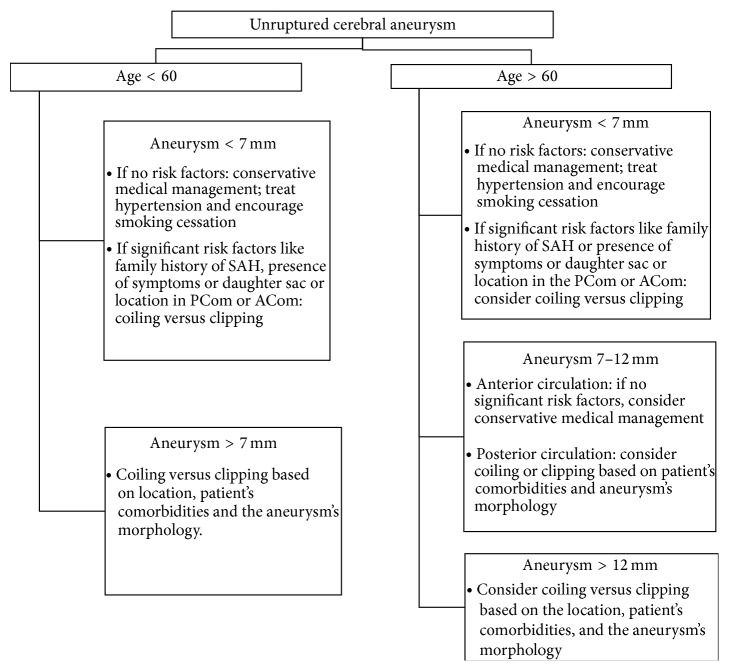
Flow chart for the management of unruptured cerebral aneurysm.

In general, microsurgical clipping is used for young patients (<50) with small aneurysms (<10 mm) in the anterior circulation unless such a patient has significant medical comorbidities that may increase their surgical risk. Moreover, clipping may be favored over coiling in some wide-necked aneurysms or aneurysms with branches arising from the neck or body. Furthermore, endovascular coiling is ideal for patients with several medical comorbidities with increased surgical risk or patients with aneurysms that have narrow necks. Aneurysms with wide necks may be coiled using stent assisted coiling or balloon angioplasty assisted coiling. Pipeline embolization (Covidien, Irvine, CA) is ideal for carotid cavernous aneurysms as well as large and giant internal carotid artery aneurysms (readers should refer to Table 5 and Figure 1).

## 8. Conclusion

Unruptured intracranial aneurysms are currently being detected at a higher rate because of increased use of imaging techniques. Once they are detected, we need to compare the aneurysm's natural history to the risk of intervention while considering the aneurysm location, size, morphology, age, clinical presentation, and medical comorbidities. These are factors that will also determine the type of interventional technique to pursue if the patient is a candidate for intervention. After an exhaustive review of the literature, the optimum management of small unruptured intracranial aneurysm remains unclear. Currently, we do not have well defined risk of rupture specific to aneurysmal size or location. Furthermore, we need randomized clinical trial that will directly compare clipping with coiling of unruptured aneurysm. Moreover, we need more data on evolving endovascular techniques like flow diverting stents.

## Figures and Tables

**Table 1:** PHASES aneurysm risk score [11].

Population	
North American or European (except Finnish)	0 point
Japanese	3 points
Finnish	5 points
Hypertension	
No	0 point
Yes	1 point
Age	
Less than 70 years	0 point
Greater than or equal to 70 years	1 point
Size of aneurysm	
Less than 7.0 mm	0 point
7.0 mm–9.9 mm	3 points
10.0 mm–19.9 mm	6 points
Greater than or equal to 20 mm	10 points
Earlier subarachnoid hemorrhage from another aneurysm	
No	0 point
Yes	1 point
Site of aneurysm	
Internal carotid artery	0 point
Middle cerebral artery	2 points
Others like anterior cerebral artery, posterior communicating	4 points
artery, or posterior circulation aneurysms	
PHASES risk score	5-year risk of aneurysm rupture
Less than or equal to 2	0.4%
3	0.7%
4	0.9%
5	1.3%
6	1.7%
7	2.4%
8	3.2%
9	4.3%
10	5.3%
11	7.2%
Greater than or equal to 12	17.8%

**Table 2:** Summary of large studies evaluating the natural history of unruptured cerebral aneurysms.

Study design	Number of aneurysms and patients	Follow-up duration in years	Predictors of cerebral aneurysm rupture	Design flaws
International Study of Unruptured Intracranial Aneurysms (ISUIA) [12]: Retrospective Study in North America and Europe	1937 unruptured aneurysms in 1449 patients	8.3	(1) Size > 10 mm (2) Posterior circulation and posterior communicating artery aneurysms	(1) Selection bias

International Study of Unruptured Intracranial Aneurysms (ISUIA) [12]: Prospective Observational Study in North America and Europe	2686 unruptured aneurysms in 1692 patients	4.1	(1) Size > 7 mm (2) Previous subarachnoid hemorrhage from some other aneurysm (3) Posterior circulation and posterior communicating artery aneurysms	(1) Selection bias (2) Follow-up was <5 years for >50% of patients

Comprehensive Observational Cohort Study by Juvela and colleagues in Finland [13]	181 unruptured aneurysms in 142 patients	21	(1) Patient's age (inversely) (2) Cigarette smoking (3) Size > 10 mm	(1) Small total number of patients (2) Homogeneity of the study population

The Unruptured Cerebral Aneurysm Study of Japan (UCAS): Prospective Cohort Study by Morita et al. [14]	6697 unruptured aneurysms in 5720 patients	Not available	(1) Size > 7 mm (2) Location: anterior and posterior communicating arteries (3) Presence of daughter sac	(1) Selection bias (2) Homogeneity of the study population

**Table 3:** Summary of large studies evaluating the microsurgical clipping of unruptured cerebral aneurysms.

Study	Important findings
International Study of Unruptured Intracranial Aneurysms (ISUIA) [12, 15]	Overall, morbidity and mortality were the highest in patients older than age 50 years and with aneurysms that were large or in the posterior circulation. In a cohort of 1917 prospectively evaluated patients, combined morbidity and mortality at 1 year was 12.6% for those without prior hemorrhage (death was 2.7%; functional disability was 1.4%; impaired cognitive status was 5.5%) and 10.1% for those with previous subarachnoid hemorrhage from some other aneurysm (death was 0.6%; functional disability was 0.9%; impaired cognitive status was 7.1%)

Britz et al. [16]	Surgical clipping in 4619 patients was associated with higher survival estimates (hazard rate of death 30%) and low neurologically related causes of death (2.3%)

Ogilvy and colleagues at Massachusetts General Hospital [17]	Treatment of 604 unruptured aneurysms showed an overall morbidity and mortality of 15.9% and 0.8%, respectively. Treatment risk for large aneurysms was 5% in the anterior versus 15% in the posterior circulation in the elderly, while treatment risk was 2% in young patients with aneurysmal size <10 mm

Moroi and colleagues at the Research Institute for Brain and Blood Vessels [18]	Treatment of 549 unruptured aneurysms showed a mortality and morbidity of 0.0% and 0.6% for aneurysms <10 mm and a mortality and morbidity of 1.2% and 6.1% for aneurysms >10 mm

Meta-analysis using Cochrane Database by Kotowski et al. [19]	Analysis of 60 studies (from 1990 to 2011) with 9845 patients with 10,845 aneurysms showing a mortality rate of 1.7% and an overall morbidity rate of 6.7%. Significant risk factors for poor surgical prognosis included aneurysm size >10 mm and posterior circulation aneurysms (*P* < 0.001)

**Table 4:** Summary of large studies evaluating the endovascular management of unruptured cerebral aneurysms.

Study	Important findings
International Study of Unruptured Intracranial Aneurysms (ISUIA) [12]	The 1-year morbidity rate was 6.4% and the mortality rate was 3.1% in 451 patients treated with endovascular coiling. The risk of poor outcome with endovascular procedure was higher with aneurysm diameter greater than 12 mm and posterior circulation location

Meta-analysis by Naggara et al. [20]: retrospective analysis of 97 studies from 2003 to 2011 with 7172 patients	Mortality rate of 1.8% and overall unfavorable outcomes rate (including death) of 4.7%. Endovascular treatment became safer over time with reduction in the rate of poor outcomes from 5.6% before 2000, 4.7% between 2001 and 2003, and 3.1% after 2004. Risk of unfavorable outcomes was 4.9% with coil embolization, 8.1% with liquid embolization agents, and 11.5% with flow diversion

Analysis of treatment by endovascular approach of nonruptured aneurysm (ATENA) by Pierot et al. [21]: prospective study on 739 unruptured aneurysms (<15 mm) treated in 27 centers in Canada and France	Morbidity and mortality at 1 month were 1.7% and 1.4%, respectively. Complications included intraoperative rupture rate of 2.6%, device-related complication rate of 2.9%, and thromboembolism rate of 7.1%

Murayama and colleagues [22]: retrospective study of 916 unruptured aneurysms treated with coil embolization	Rate of recanalization was 20.9%. The recanalization rate for small aneurysms (<10 mm) with narrow necks (<4 mm) was 5.1%, whereas, in small aneurysms with wide necks (>4 mm), it was 20.0%. The recanalization rate was 35.0% in large aneurysms (11–25 mm) and 59.1% in giant aneurysms (>25 mm)

Benes and colleagues [23]: analysis of 151 unruptured aneurysms treated with coil embolization in 131 patients	Combined morbidity and mortality rate of 1.5% at 6 months. Thromboembolic complication rate of 7.6%

**Table 5:** Summary of large studies evaluating the treatment risk of unruptured cerebral aneurysms.

Study	Important findings
Alshekhlee et al. [24]: review of a cohort of 3,738 clipped unruptured aneurysms versus 3,498 coiled unruptured aneurysms from the National Inpatient Sample Database from 2000 to 2006	(i) Mortality rate was 1.61% (for clipped aneurysms) versus 0.57% for coiled aneurysms (*P* < 0.0001) (ii) Rate of acute ischemic stroke was 6.71% (for clipped aneurysms) versus 2.92% for coiled aneurysms (*P* < 0.0001) (iii) Rate of intracerebral hemorrhage was 2.38% (for clipped aneurysms) versus 1.37% for coiled aneurysms (*P* < 0.002)

McDonald et al. [25]: review of a cohort of 1,388 clipped unruptured aneurysms versus 3,551 coiled unruptured aneurysms from Premier Perspective Database from 2006 to 2011	(i) Mortality rates were similar in both clipping and coiling with odds ratio of 1.43 (*P* < 0.47) (ii) Clipping had a higher likelihood of unfavorable outcomes: odds ratio (OR) for discharge to long term care was 4.78 (*P* < 0.0001); OR for ischemic complications was 3.42 (*P* < 0.0001); OR for postoperative neurological complications was 3.39 (*P* < 0.0001); OR for hemorrhagic complications was 2.16 (*P* < 0.0001); OR for ventriculostomy was 2.10 (*P* < 0.032)

## Abbreviations

CTA: CT angiography
ISUIA: International Study of Unruptured Intracranial Aneurysms
MRA: Magnetic resonance angiography
SAH: Subarachnoid hemorrhage.
